# Mutation in Archain 1, a Subunit of COPI Coatomer Complex, Causes Diluted Coat Color and Purkinje Cell Degeneration

**DOI:** 10.1371/journal.pgen.1000956

**Published:** 2010-05-20

**Authors:** Xinjie Xu, Rajendra Kedlaya, Hitoshi Higuchi, Sakae Ikeda, Monica J. Justice, Vijayasaradhi Setaluri, Akihiro Ikeda

**Affiliations:** 1 Department of Medical Genetics, University of Wisconsin–Madison, Madison, Wisconsin, United States of America; 2 Department of Dermatology, University of Wisconsin–Madison, Madison, Wisconsin, United States of America; 3 Department of Molecular and Human Genetics, Baylor College of Medicine, Houston, Texas, United States of America; Stanford University School of Medicine, United States of America

## Abstract

Intracellular trafficking is critical for delivering molecules and organelles to their proper destinations to carry out normal cellular functions. Disruption of intracellular trafficking has been implicated in the pathogenesis of various neurodegenerative disorders. In addition, a number of genes involved in vesicle/organelle trafficking are also essential for pigmentation, and loss of those genes is often associated with mouse coat-color dilution and human hypopigmentary disorders. Hence, we postulated that screening for mouse mutants with both neurological defects and coat-color dilution will help identify additional factors associated with intracellular trafficking in neuronal cells. In this study, we characterized a mouse mutant with a unique N-ethyl-N-nitrosourea (ENU)–induced mutation, named *nur17*. *nur17* mutant mice exhibit both coat-color dilution and ataxia due to Purkinje cell degeneration in the cerebellum. By positional cloning, we identified that the *nur17* mouse carries a T-to-C missense mutation in archain 1 (*Arcn1*) gene which encodes the δ subunit of the coat protein I (COPI) complex required for intracellular trafficking. Consistent with this function, we found that intracellular trafficking is disrupted in *nur17* melanocytes. Moreover, the *nur17* mutation leads to common characteristics of neurodegenerative disorders such as abnormal protein accumulation, ER stress, and neurofibrillary tangles. Our study documents for the first time the physiological consequences of the impairment of the ARCN1 function in the whole animal and demonstrates a direct association between ARCN1 and neurodegeneration.

## Author Summary

The fact that mutations in single gene can result in defects in multiple organs suggests the existence of similar fundamental cellular mechanisms shared by different organs. Thus, genes that are important for a certain cellular function in a tissue of interest can be identified by searching for mutations that cause visible abnormalities indicative of such defect in other tissues. Here, we identified a gene essential for vesicle trafficking in neuronal cells by screening for mouse axtaxia mutants with diluted coat color, which often indicates defective intracellular trafficking in skin cells. This mouse mutant, neurological 17 (*nur17*), was generated by ENU mutagenesis. By positional cloning, we identified a mutation in the archain 1 gene encoding a component of the COPI complex, which is known to be involved in intracellular protein trafficking. The mutation in archain 1 results in phenotypes commonly observed in neurodegenerative disorders such as abnormal protein accumulation, ER stress, and neurofibrillary tangles. *nur17* mice provide a new model to study the association between intracellular trafficking and the mechanisms of neurodegenerative diseases.

## Introduction

Intracellular trafficking is critical for delivering molecules and organelles to their proper destinations to perform normal cellular functions (reviewed in [1]). Proteins and lipids are transported to the target cellular compartments through vesicular trafficking pathways, and organelles are trafficked along microtubules and actin cytoskeleton. Impairment of intracellular trafficking has been implicated in the pathogenesis of various neurodegenerative disorders, such as Alzheimer's disease (reviewed in [2], [3]), Huntington disease [4]–[6] and Parkinson's disease [7], indicating the importance of intracellular trafficking for proper function and maintenance of neuronal cells. However, factors that are involved in this process and their associations with the mechanisms causing neurodegeneration are not yet fully understood.

The molecular mechanisms of intracellular trafficking are shared by different cell types and organ systems. This is evident from the fact that multiple organ systems are affected in a number of human diseases caused by defects in intracellular trafficking. For example, Hermansky-Pudlak Syndrome (HPS), a collection of heterogeneous genetic disorders caused by defects in intracellular vesicle trafficking, is characterized by oculocutaneous albinism and defective platelet storage [8], suggesting that the affected protein trafficking pathways are shared between skin and blood cells. Fundamental intracellular trafficking mechanisms are also shared by the skin and nervous systems. For example, Griscelli syndrome (GS) type 1 which is caused by mutations in the myosin 5a (*MYO5A*) gene is characterized by pigmentary dilution of the skin and hair and neurological defects with severe ataxic movement [9]. The *Myo5a* mouse mutant, *dilute*, also exhibits coat color dilution and severe ataxic movement [10]. Studies on *dilute* mice demonstrated that MYO5A, an actin-based motor protein, participates in organelle trafficking in both melanocytes and neuronal cells [11]–[13]. Intracellular melanosome trafficking is disrupted in melanocytes of *dilute* mice, leading to coat color dilution [13]. Moreover, endoplasmic reticulum (ER) transport in cerebellar Purkinje cells (PCs) is disrupted in *dilute* mutants, which may result in neurological defects [11]. Mutations in *Fig4* and *Vac14*, affecting the conversion of phosphatidylinositol-3-phosphate (PI3P) to the signaling lipid phosphatidylinositol-3,5-bisphosphate (Pl(3,5)P_2_), also lead to neurodegeneration and diluted pigmentation in mice [14], [15]. Pl(3,5)P_2_ regulates vesicle trafficking in the endosome-lysosome axis in yeast [16], and abnormalities indicative of defects in the regulation of endosomal vesicles are observed in these mutant mice [14], [15]. Pigment containing hair follicles are greatly reduced in the skin of *Fig4* mutant mice, suggesting that lysosome-melanosome biogenesis may be affected [14]. Another mouse mutant with both pigmentary and neurological defects is the sandy mouse with a mutation in a component of biogenesis of lysosome-related organelles complex 1 (BLOC-1), which regulates trafficking to lysosome-related organelles including melanosomes [17].

Based on the findings that the vesicle/organelle trafficking pathways are shared among multiple organs, we postulated that screening for mouse mutants exhibiting phenotypes indicative of defects in intracellular trafficking in multiple tissues may yield molecules important for this process. Among defects in different organs, coat color dilution is a good indicator for defective intracellular trafficking because the defect can be readily observed in the whole animal. A number of mouse mutants that show coat color dilution have been isolated and defective vesicle and organelle trafficking pathways are the major causes of coat color dilution in these mice [8], [18]. For example, all the sixteen mouse mutants with dilute coat color that were identified as models to study HPS carry mutations in genes that are involved in vesicle trafficking to melanosomes [8], [19], [20]. Mouse models of Griscelli syndrome also exhibit dilute coat color and bear mutations in components of the RAB27A-MLPH-MYO5A complex that are necessary for proper intracellular trafficking of melanosomes [10], [21], [22].

In this study, we screened for mice with both a neurological defect (ataxia) and coat color dilution, and identified a novel mutant, neurological 17 (*nur17*). We performed positional cloning of the *nur17* mutation and identified a single nucleotide substitution in archain 1 (*Arcn1*), a highly conserved gene that encodes the δ subunit of the coat protein I (COPI) complex [23]. Further characterization of the coat color and neuronal phenotypes indicated that the *nur17* mutation perturbs intracellular protein trafficking and ER function in the affected tissues. Our study is the first demonstration of the physiological consequences of the impairment of the ARCN1 function in mammalian tissues *in vivo*, and provides a direct link between the ARCN1 functions and neurodegeneration.

## Results

### Coat-color dilution and progressive Purkinje cell degeneration in *nur17* mice

**Figure 1:**
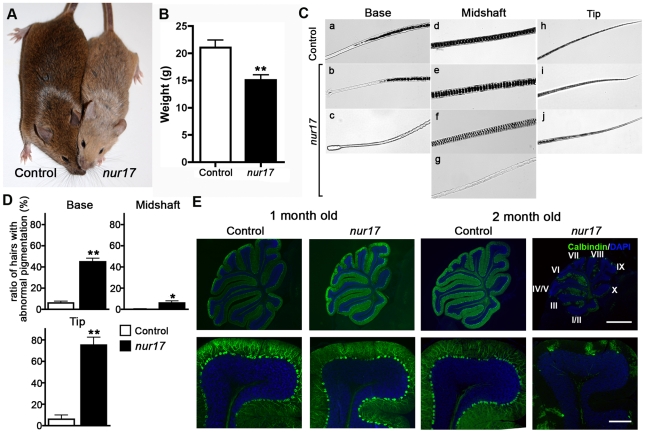
Coat color dilution and Purkinje cell degeneration in *nur17* mice. (A) Diluted coat color in *nur17* mice (right) compared with control mice (left). (B) Body weight of 2-month old *nur17* (n = 7, female) and control (n = 5, female) mice. Error bars represent standard error. **p<0.01 (C) Pigment distribution in the hair of control and *nur17* mice. Base, midshaft and tips of the hair of control (top) and *nur17* (bottom) mice are shown. Representative pictures from 30 hairs of each genotype are shown. (D) Ratio of hairs with abnormal pigmentation in the base (left), midshaft (center) and tip (right). Total of 150 hairs from 5 *nur17* and 5 control mice (30 hairs each) were examined. Error bar represent standard error. *p<0.05; **p<0.01 (E) Degenerative loss of the Purkinje cells in *nur17* mice. Purkinje cells are labeled with anti-calbindin (green) and nuclei are stained with DAPI (blue). Note that by 2 months of age, *nur17* mice lose their Purkinje cells in Lobule IX. Upper scale bar: 1mm; Bottom scale bar: 0.1mm.

The autosomal recessive *nur17* mutation was generated by ENU mutagenesis at Mouse Mutagenesis Center for Developmental Defects at Baylor College of Medicine [24], [25]. Initial phenotypic characterization showed that *nur17* mice exhibit both coat color dilution (Figure 1A) and ataxic movements. The coat color dilution phenotype of *nur17* mice was noted to be milder compared with that of *dilute-lethal* (*Myo5a^d-l^*) mice [10], and to become more notable with age. The ataxic phenotype, which is also milder in *nur17* mice than that of *Myo5a^d-l^* mice, was observed around the age of 2 months and developed progressively as the mice age. The size of *nur17* mice was smaller compared to unaffected control littermates (+/+ or *nur17*/+) (Figure 1A and 1B) (control: n = 5, *nur17* mice: n = 7; p<0.01 by student's t-test).

To further characterize the coat color dilution, we examined the distribution of pigment in hair samples from *nur17* and littermate control (+/+, *nur17*/+) mice. In the hair of control mice, the pigment was incorporated all the way to the tip in a regularly repeated pattern (Figure 1C, top). In the hair of *nur17* mice, we observed increased spacing between pigment bands and an occasional absence of pigment in the midshaft (Figure 1C, bottom). The ratio of hairs with abnormal pigmentation at the base, midshaft and tip was significantly higher in *nur17* mice compared to control mice (control: n = 5, *nur17* mice: n = 5, 30 hairs from each mouse, Student's t-test, Figure 1D). These hair phenotypes suggest that the *nur17* gene product is required for the proper transport/incorporation of pigment into the hair.

*nur17* mice also exhibit ataxic movements beginning around 2 months of age. Because the cerebellum is the center for motor coordination, we examined the morphology of the cerebellum in *nur17* mice. Purkinje cells (PCs) were visualized using a PC marker, anti-calbindin [26]. In littermate control (+/+, *nur17*/+) mice, PCs were aligned in a single cell layer adjacent to each other (Figure 1E). At 1 month of age before the appearance of ataxic movements, *nur17* mice showed normal cerebellar morphology with an intact PC layer despite showing a few signs of degeneration (Figure 1E). However, in 2-month-old *nur17* mice, we observed extensive loss of PCs compared to control mice (Figure 1E, right panels). We also found a specific regional pattern for PC degeneration in *nur17* mice. By 2 months of age, most of the PCs in a part of lobule VI and lobules VII to X of *nur17* mice have degenerated (Figure 1E). In contrast, PCs in lobules I to V remained at this age (Figure 1E).

### Positional cloning of the *nur17* gene

**Figure 2:**
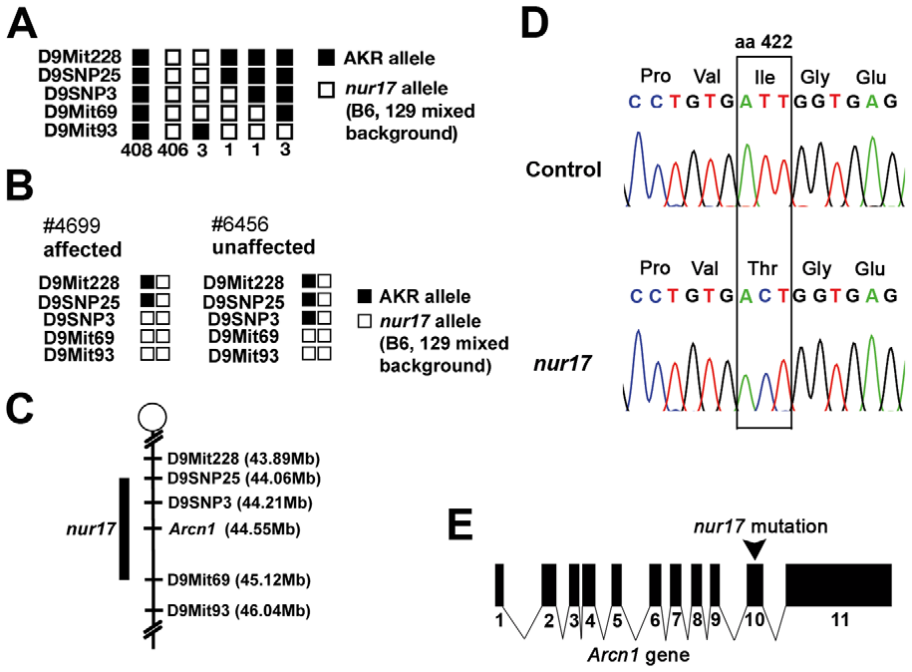
Positional cloning of the *nur17* gene. (A) The haplotypes for 822 chromosomes from 411 F2 mice are indicated. (B) Genotypes and phenotypes of two recombinant mice that were critical for determining the minimal region for the *nur17* locus. (C) Minimal genetic region of the *nur17* locus. (D) Point mutation in the *Arcn1* gene from *nur17* mice. T to C nucleotide transition causes an amino-acid change from Ile to Thr at amino acid (aa) 422. (E) Point mutation is located in the 10^th^ exon of the *Arcn1* gene in *nur17* mice.

We genetically mapped the *nur17* gene on mouse chromosome 9 using an F2 intercross (*nur17 *× AKR). All F1 animals (14 females and 13 males) were phenotypically normal and did not show any coat color dilution or ataxic phenotypes. To map the *nur17* locus, we employed microsatellite markers and single-nucleotide polymorphisms (SNPs) to distinguish between the alleles of *nur17* (mixed background of C57BL/6J, 129S6/SvEvTac and 129S1/SvImJ) and AKR. For initial mapping, we tested genomic DNA from F2 mice including 8 affected mice with 80 microsatellite markers across the whole genome. We observed co-segregation of a marker D9Mit69 with the *nur17* phenotypes and found no significant linkages with other chromosomal loci. Additional F2 animals were tested to further narrow down the *nur17* locus. We collected a total of 822 meioses (411 F2 mice) (Figure 2A). Of note, the coat color dilution and ataxic phenotypes always co-segregated in this mapping cross, indicating that a single gene mutation likely accounts for both phenotypes. After progeny testing of F2 mice carrying critical recombination (Figure 2B), the minimal genetic region of *nur17* was determined to be between *D9SNP25* and *D9Mit69* (Figure 2C). A total of 36 genes are localized within this 1.06 Mb interval. We selected 4 genes, dolichyl-phosphate N-acetylglucosamine phosphotransferase (*Dpagt1*), vacuolar protein sorting 11 (*Vps11*), trafficking protein particle complex 4 (*Trappc4*) and archain 1 (*Arcn1*), as candidates for *nur17*, since they are known to be associated with vesicle trafficking. We sequenced the coding regions of these candidate genes in *nur17* mice and did not observe any nucleotide change in *Dpagt1*, *Vps11* and *Trappc4*. However, we identified a single nucleotide change in the *Arcn1* gene (Figure 2D). This T to C conversion in the 10^th^ exon of the *Arcn1* gene causes an amino acid change from isoleucine (Ile) to threonine (Thr) at amino acid 422 (Figure 2D and 2E). The mutation is in the vicinity of the cargo-binding site of ARCN1 which recognizes arginine-based ER localization signals [27].

### Transgenic expression of wild-type *Arcn1* rescues *nur17* phenotypes

**Figure 3:**
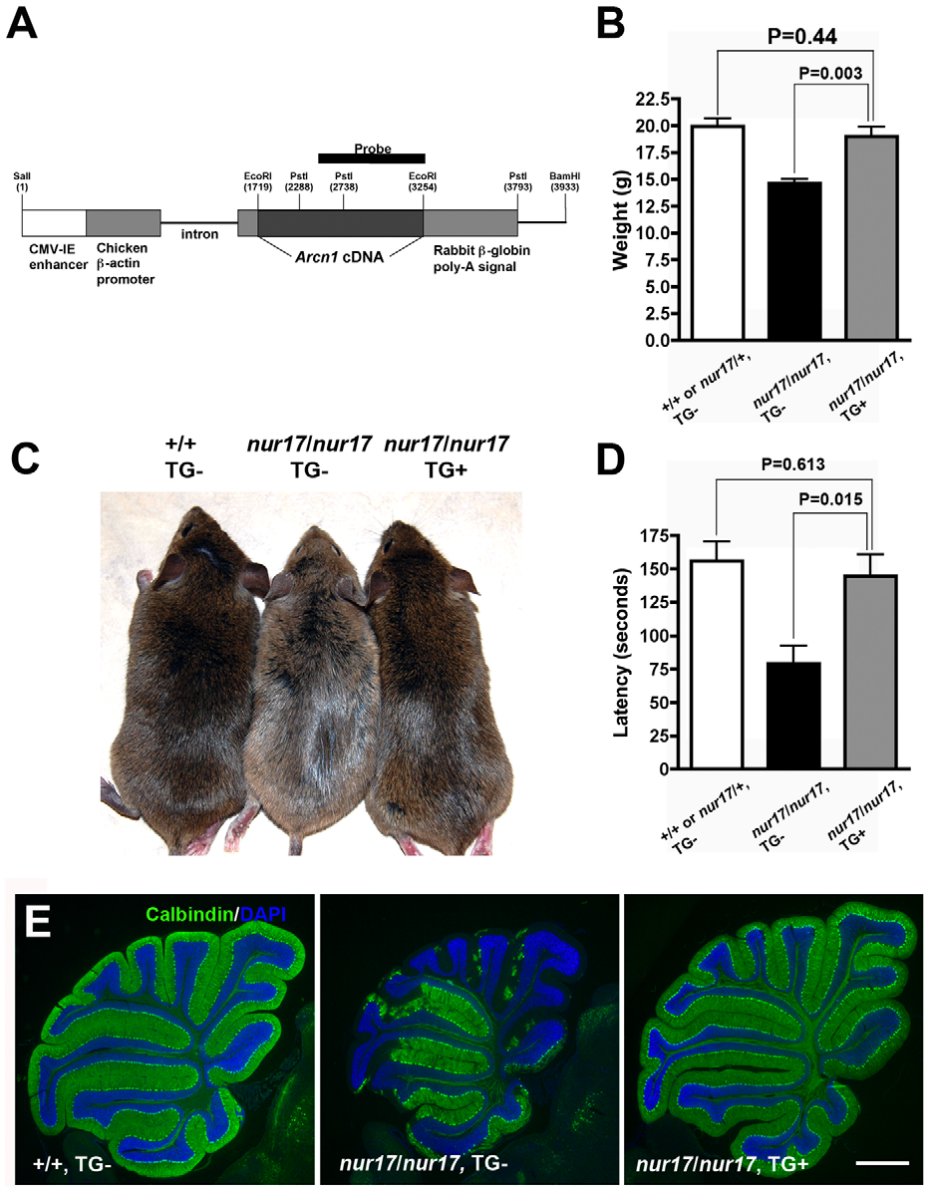
Phenotypes of *nur17* mice are rescued by wild-type *Arcn1*. (A) Transgenic construct for generating the transgenic mice expressing *Arcn1* gene. Restriction enzyme (*Pst*I) sites to digest DNA for southern blotting were labeled. The black bar represents the region used for the probe for southern blot. (B) Rescue of smaller body weight by transgene in *nur17* mice. Smaller size in *nur17* mice (middle, n = 13, female) is rescued by the introduction of transgene, *Arcn1* (right, n = 6, female) (p = 0.003). There is no significant difference between the weight of rescued mice (right) and the control mice (left, n = 7, female) (p = 0.44). (C) Rescue of coat color dilution. Coat color dilution phenotype in *nur17* mice (middle) is rescued by the introduction of transgene, *Arcn1* (right). (D) Rescue of the motor coordination defect. Latency to fall on the accelerating rotatod was recorded for control mice (n = 5), *nur17* mice (n = 5) and *nur17* mice with the *Arcn1* transgene (n = 5). The rotarod performance was significantly improved by the transgene (p = 0.015), and was comparable to that in control mice (p = 0.613). (E) Rescue of Purkinje cell degeneration. Purkinje Cell degeneration phenotype in 2-month old *nur17* mice (middle) is completely rescued by the introduction of transgene, *Arcn1* (right). Purkinje cells are labeled with anti-calbindin (green) and nuclei are stained with DAPI (blue). Scale bar: 1mm.

To confirm that the *Arcn1* mutation is responsible for the phenotypes in *nur17* mice, we tested whether transgenic (Tg) expression of wild-type *Arcn1* can rescue the *nur17* phenotypes. We generated a Tg construct (Figure 3A), in which expression of the *Arcn1* cDNA is driven by the chicken β-actin promoter and is followed by the rabbit β-globin poly-A sequence (Figure 3A) [28]. We obtained 2 founder mice (2361 and 2368) that carried the transgene and named the mice generated from these 2 founder mice as line 2361 and line 2368 (Figure S1A). Phenotypes of *nur17* mice were rescued in line 2361, in which expression of the transgene was confirmed (Figure 3B–3D, Figure S1B). The coat color dilution and lower body weight in *nur17* mice were completely rescued in mice expressing the wild-type *Arcn1* transgene (Figure 3B and 3C). We also analyzed the ataxic phenotype using rotarod (Figure 3D). While this analysis revealed significantly decreased latency in *nur17* mice compared to control mice (p = 0.0046 by student's t-test), this phenotype was rescued by the expression of the *Arcn1* transgene (Figure 3D). In addition, we examined the PC degeneration phenotype in 2 month-old mice and observed no PC degeneration in *nur17* mice carrying the transgene (Figure 3D, right). We tested a total of 7 *nur17/nur17*;*Tg/+* mice and the phenotypes were rescued in all of these mice. In line 2368, the phenotypes of *nur17* mice were not rescued (data not shown). However, we found that the transgene is not expressed in this line (Figure S1B). These results demonstrated that the single nucleotide change in *Arcn1* is the causal mutation in *nur17* mice.

### Expression and cellular localization of ARCN1

**Figure 4:**
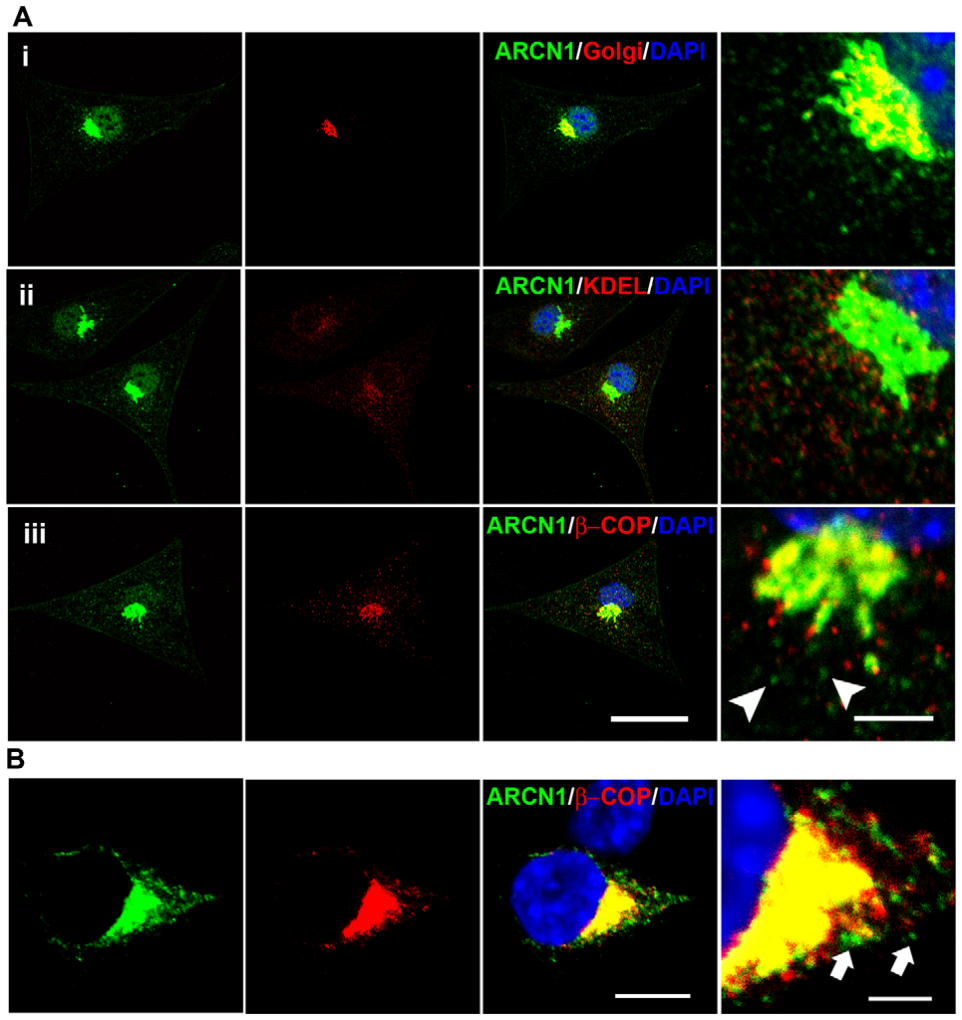
Subcellular localization of ARCN1. (A) Primary culture of wild-type mouse melanocytes are labeled with anti-ARCN1 (green) indicating the localization of ARCN1 and anti-Golgi protein (red) (i); anti-KDEL (red) (ii) and anti-βCOP (red) (iii). (B) Neuro-2a cells transfected with constructs for GFP-tagged ARCN1 (green) and V5-tagged βCOP (red). Nuclei are stained with DAPI (blue). Merged pictures are shown and high magnification pictures are shown in the right panel. Scale bar for low magnification: 10µm. Scale bar for high magnification: 5µm.

ARCN1, also known as δ-COP, is a subunit of the coat protein I (COPI) complex [29]. The COPI complex was first isolated from mammalian sources as a cytosolic protein complex and components of Golgi derived vesicles [30], [31]. It was originally found to be involved in retrograde trafficking from the cis-Golgi to the rough ER (reviewed in [32]) and in intra-Golgi trafficking (reviewed in [33], [34]) mostly based on studies in yeast. Studies using mammalian cells with a mutation or depletion of a COPI subunit have shown that COPI is essential for compartmentalization of secretory compartments, proper Golgi structure, ER-Golgi transport and endosome functions [35]–[38]. Although the phenotypes of a mutant for the yeast homolog of ARCN1 suggests its involvement in both retro and antero-grade ER-Golgi trafficking [39], the role of this particular COPI subunit (δ-COP) in mammalian cells and the physiological consequences of its impairment in the whole animal have not been examined. In order to understand the function of ARCN1 in mammalian cells, we first examined sub-cellular localization of ARCN1 using anti-ARCN1 antibody (green) in primary cultured mouse melanocytes (Figure 4). ARCN1 was co-localized with Golgi (Figure 4Ai) and ER (Figure 4Aii) markers (red) and showed a punctate staining pattern in the cytoplasm suggesting its localization in the vesicular compartment (Figure 4Ai and Aii). In mouse melanocytes, ARCN1 was co-localized with β-COP (red, Figure 4Aiii), which is a known binding partner of ARCN1 in yeast [29], mainly in ER and Golgi. This result indicated that ARCN1 (δ-COP) is present in a complex with β-COP in mammalian cells. We also noted that there are vesicles positive for only ARCN1 (green, arrowheads) and not β-COP (red) (Figure 4Aiii). This observation was confirmed by co-expressing GFP-tagged ARCN1 and V5-tagged β-COP (COPB1) in Neuro-2a cells (Figure 4B). The ARCN1 signal does not always co-localize with β-COP signal. These results indicate that ARCN1 may also exist unbound to β-COP in mammalian cells, suggesting the possibility that ARCN1 may also have a function independent of β-COP or the COPI complex. Further biochemical studies are required to test these possibilities.

### ARCN1 is involved in ER–Golgi and/or intra-Golgi trafficking in melanocytes

**Figure 5:**
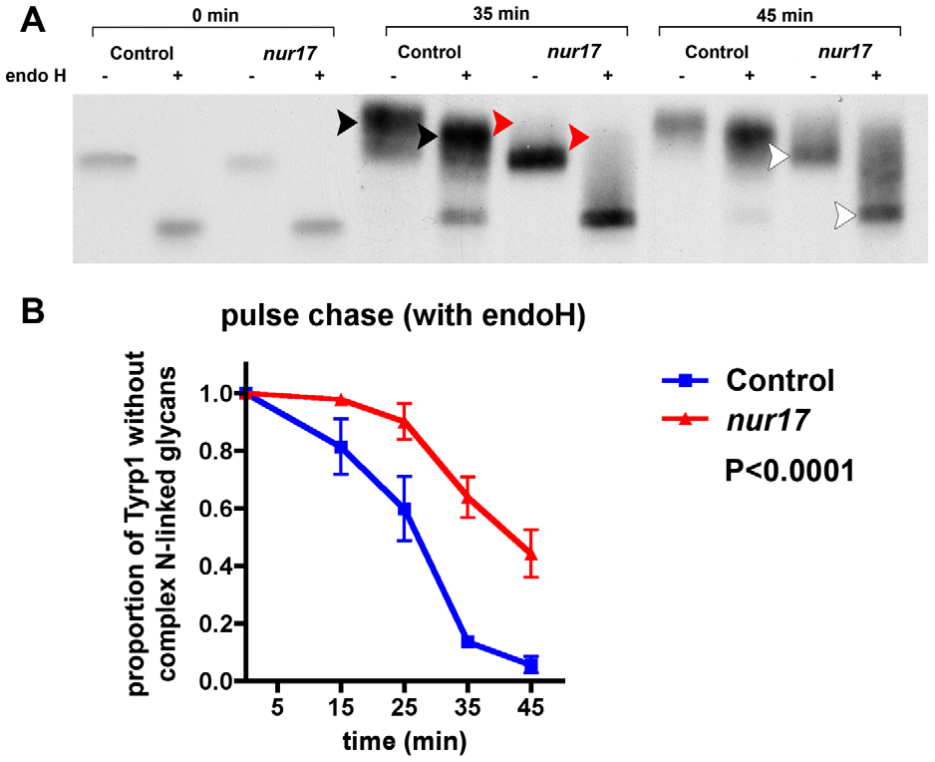
Kinetic analysis of biosynthesis and transport of Tyrp1 in the melanocyte. (A) Pulse chase analysis of control and *nur17* melanocytes. Control and *nur17* melanocytes were pulse labeled for 10 min with [^35^S] methionine followed by a chase as the indicated times. The size of Tryp1 is monitored after pulse labeling with [^35^S] and analyzed by 9% SDS-PAGE. (Molecular weight markers are shown on the left.) (B) Quantification of the proportion of Tyrp1 without complex N-linked glycans at 5 different chasing time points (0min, 15min, 25min, 35min and 45min). Error bar represents standard error.

In order to examine protein trafficking through ER and Golgi in *nur17* cells, we performed pulse-chase analysis in *nur17* melanocytes using the melanosomal membrane protein Tyrp1 as an indicator for intracellular trafficking [40], [41]. We monitored ER-Golgi transport using endoglycosidase H (endo H), which cleaves N-linked sugar chain moieties on newly synthesized proteins that are localized in the ER or intermediate compartments but not those that have been modified in the Golgi. Without endo H treatment [endo H (−)], newly synthesized Tyrp1 molecules that have been glycosylated during biosynthetic transport through ER and Golgi are represented by bands with higher molecular weight compared to the band in the endo H (−) lane at 0 min. With endo H treatment [endo H(+)], sugar chains on Tyrp1 molecules that have been processed in Golgi and thus become resistant to endo H digestion are represented by bands with higher molecular weight compared to the band in the endo H (+) lane at 0 min. In control melanocytes (from +/+, *nur17*/+ littermates), after 35 minutes of chase, higher molecular weight bands were observed with endo H and without endo H (black arrowheads in Figure 5A), suggesting that some of the Tyrp1 molecules have been trafficked into Golgi and undergone processing that confers endo H resistance. In contrast, little or no higher molecular weight bands were observed in the *nur17* melanocytes at this timepoint (red arrowheads in Figure 5A). At 45 min, the bulk of Tyrp1 protein was shifted to a higher molecular weight form in control melanocytes, whereas a band of lower molecular weight still remained in *nur17* melanocytes (white arrowheads). We quantified this shift by measuring the intensity of radioactive signals in these bands as shown in Figure 5B. The proportion of protein without complex N-linked glycans (lower band) was significantly higher in melanocytes of *nur17* mice compared to cells from control mice (P<0.0001, 2 way ANOVA). These results suggest that the efficiency of protein trafficking through ER and Golgi may be affected in *nur17* mice. Alternatively, it is possible that these results indicate pertubation of the glycosylation process in the Golgi of *nur17* mice. If retrograde transport of oligosaccharide transferases within the Golgi apparatus is defective due to the mutation, deprivation of these enzymes in appropriate Golgi cisternae could occur, causing delay in glycosylation. This scenario would be consistent with the proposed function of COPI in intra-Golgi retrograde transport of resident proteins in the cisternal maturation model of protein transport through Golgi [34].

### ER stress and abnormal protein aggregation in *nur17* mice

**Figure 6:**
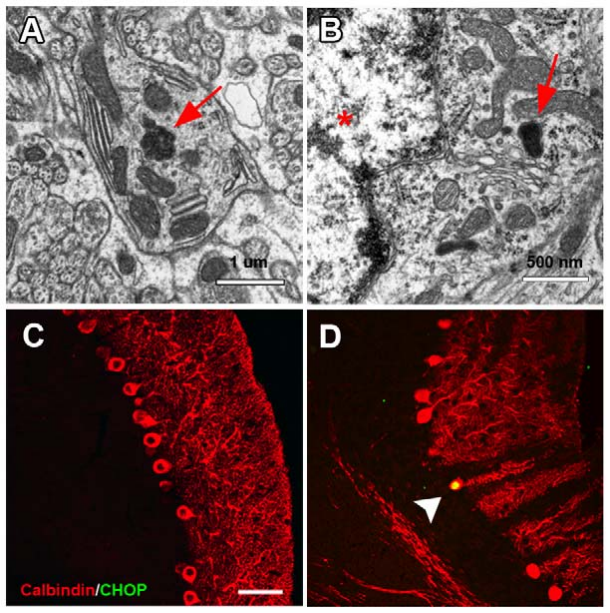
Abnormal protein aggregation and upregulated ER stress marker in the cerebellum of *nur17* mice. (A) Ultrastructural analysis showed protein inclusion (red arrow) in the dendrite of PC. (B) Protein inclusion (red arrow) also exists in the perinuclear region of PC. PC nucleus is marked by a red star. (C, D) PC labeled with anti-Calbindin (red) is negative for anti-CHOP antibody staining (green) in control mice (C) but positive in *nur17* mice (D, white arrowhead). Scale bar: 30um.

Based on the possible trafficking defects observed in melanocytes of *nur17* mice, we hypothesized that such intracellular trafficking mechanism is also impaired by the *nur17* mutation in the PC. Electron microscopic analysis of the cerebellum of *nur17* mice showed abnormal accumulation of proteins in the dendrites of PCs (red arrow, Figure 6A) as well as in perinuclear areas (red arrow, Figure 6B, PC nucleus is marked by red star) [42]. Since accumulation of misfolded protein has been associated with defects in vesicle trafficking followed by ER stress [7], we then examined whether ER stress occurs in the degenerating PCs of *nur17* mice using an ER stress marker, anti-CCAAT/enhancer-binding protein homologous protein (CHOP) [42]. We observed high levels of CHOP signals in the cell bodies of some PCs in *nur17* mice (Figure 6D, white arrowhead), while we never observed CHOP signals in PCs of littermate control (+/+, *nur17*/+) mice (Figure 6C). The mean ratio of CHOP positive PCs against total PCs was statistically higher in *nur17* mice (0.84%; n = 4) compared to control mice (0%; n = 3) (p = 0.016 by student's *t*-test). The fact that not all PCs were positive for CHOP staining is consistent with the idea that PC degeneration does not occur simultaneously in all PCs but rather occurs progressively. We also performed immunohistochemistry with an anti-KDEL antibody, which recognizes the ER stress markers, BiP/Grp78 and Grp94. Although KDEL signals were detected in PCs of both control and *nur17* mice, no difference was observed between them (Figure S2).

### Neurofibrillary tangles in the cerebellum of *nur17* mice

**Figure 7:**
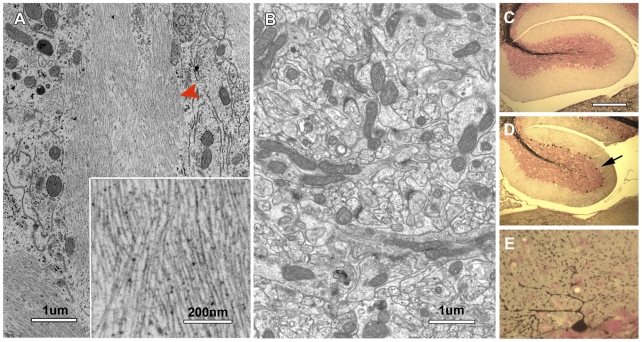
Neurofibrilary tangles in the cerebellum of *nur17* mice. (A, B) Ultrastructural analysis showed abnormal filamentous lesions (red arrowhead) in the cerebellum of *nur17* mice (A), while such lesions are not observed in control mice (B). The inset in (A) shows the higher magnification of filamentous lesion. (C–E) Gallyas staining for control (C) and *nur17* mice (D, E). Arrow denotes the PC that is positive for Gallyas staining. Scale bar for (C, D): 1mm. Scale bar for (E): 0.1mm.

Through electron microscopic analysis, we also observed abnormal filamentous lesions in the cerebellum of all 30-day-old *nur17* mice examined (n = 3) (Figure 7A), but not in the littermate control (+/+, *nur17*/+) (n = 3) mice. We examined the distribution of abnormal accumulation of filaments by Gallyas staining, which is known to stain protein aggregates of neurofilaments [43]. Central sagittal sections of the cerebellum from 4 control and 5 *nur17* mice at 4 weeks of age were subjected to Gallyas staining (Figure 7B–7D), and the number of positively-stained cells was counted. While we observed no positively-stained cells in control mice (n = 4), an average of 83±28.8 PCs in the central sagittal section of cerebellum were positively stained in *nur17* mice (n = 5, p<0.05, Student's t-test). High magnification images (Figure 7D) showed that staining is localized to the PC body and dendrites. Although hyperphosphorylated tau is known as the major component of NFTs in Alzheimer's disease, we did not observe signals when immunohistochemistry was performed on the cerebellum of *nur17* mice using anti-hyperphosphorylated tau antibodies (data not shown), indicating that NFTs in *nur17* mice may not contain hyperphosphorylated tau. It has been suggested that hyperphosphorylation of tau is not obligatory in the formation of neurofibrillary tangles [44], which may be the case in this mutant.

## Discussion

Here we show that a mutation in *Arcn1* is responsible for the phenotypes in an ENU-induced mouse mutant, *nur17*, which was identified by screening for mice with both diluted coat color and ataxia. We identified a missense mutation in *Arcn1* in *nur17* mice and confirmed that it is responsible for coat color dilution and PC degeneration by transgenic rescue. ARCN1, also known as δ-COP, is a sub-unit of the coat protein I (COPI) complex, which comprises one of the protein coats that mediate vesicle budding from the membrane (reviewed in [32], [45]–[47]). Thus, *nur17* mice provide a unique model to study the intracellular trafficking and its association with neuronal function and pigmentation in mammals.

Our results suggest that the single nucleotide substitution causing an amino acid change from isoleucine to threonine results in abnormal function of the ARCN1 protein. Since the mode of inheritance for the *nur17* mutation is recessive, the mutation was expected to cause loss of the protein function rather than gain of function. Our transgenic rescue experiment showed that the wild-type allele of *Arcn1* completely rescues the phenotypes of *nur17* mice, providing evidence for this notion. We postulate that *nur17* causes partial rather than complete loss of ARCN1 function based on the following reasons. First, *Arcn1* encodes a protein that is highly conserved from yeast to human, and mutations of *Arcn1* (δ-COP) orthologs in other species such as yeast [29], *Drosophila* (http://flybase.org, Flybase ID: FBal0096846) and *C. elegans* (http://www.wormbase.org/db, Wormbase RNAi ID: WBRNAi00033328 and WBRNAi00076118, [48]) all result in lethality, suggesting that this molecule is fundamental to cellular integrity and function. Secondly, mutants of other subunits of the COPI complex in *Drosophila* and zebrafish also result in early embryonic lethality [49], [50]. Compared to the phenotypes in other organisms, the phenotypes of *nur17* mice are rather mild, supporting the possibility that the *nur17* mutation causes partial loss of ARCN1 function. Thirdly, the mutation does not cause RNA instability or improper processing of the protein, which could cause complete loss of function, as the expression and localization of ARCN1 are not altered in the *nur17* melanocytes (Figure S3). Alternatively, it is possible that other molecules with similar functions compensate for the loss of ARCN1 function to some extent. However, there are no obvious or known molecules that could play such a role. Thus, it is likely that this missense mutation uncovers roles for COPI that would not be discovered in a complete loss of function allele.

The COPI complex was originally found to be involved in retrograde vesicle trafficking from the cis-Golgi to the rough ER (reviewed in [32]). However, studies in yeast suggested that δ-COP may be involved in both anterograde and retrograde ER-Golgi trafficking [39]. Other studies also suggested expanded roles of COPI complex in anterograde ER-Golgi trafficking and trafficking between other compartments such as early endosomes and the secretory pathway [35], [38], [51]. Consistent with these findings, our results support the possibility that δ-COP has multiple roles in vesicle trafficking. First, the *in vitro* pulse-chase assay indicated that the *nur17* mutation causes either delays in anterograde ER-Golgi protein trafficking or defects in intra-Golgi retrograde transport of resident proteins, suggesting the involvement of δ-COP in these processes in mammalian cells. Secondly, the localization study showed that ARCN1 is not only localized in ER-Golgi, but also distributed in the cytosolic space, which may represent localization of ARCN1 to other vesicular structures such as endosomes or lysosomes. Further analysis of *nur17* mice should reveal whether ARCN1 functions in these organelles and whether the mutation affects their functions. Our pulse-chase study suggested a potential link between the *Arcn1* mutation and coat color dilution. Tyrp1 is a tyrosinase related protein (TRP) and has been implicated in the biogenesis of melanosomes [52], [53]. The retardation of ER-Golgi transport of Tyrp1 from ER to Golgi in *nur17* melanocytes or impaired glycosylation of this protein could potentially delay or inhibit melanosome biogenesis resulting in coat color dilution. It is also possible that ER-Golgi trafficking of other proteins required for melanosome synthesis is affected as well. However, impaired ER-Golgi and/or intra-Golgi trafficking may not be the only cause of coat color dilution. As discussed above, the potential site of action of ARCN1 is not restricted to ER-Golgi. Since biogenesis of melanosomes includes multiple aspects of vesicle regulation such as endocytosis and recycling of vesicles (reviewed in [54]), these processes could be also affected in *nur17* melanocytes. Further analysis is needed to test these possibilities.

Defects in ER-Golgi or intra-Golgi trafficking similar to those observed in melanocytes of *nur17* mice could also be responsible for PC degeneration in the cerebellum. For example, defective ER-Golgi trafficking could be the direct cause of the formation of abnormal protein accumulation in the ER, which could in turn result in ER-stress. On the other hand, ER-Golgi trafficking is required for the unfolded protein response (UPR), which is activated by ER-stress [55]. UPR helps to restore the normal ER function by halting protein synthesis and upregulating molecular chaperones involved in protein folding and ER associated degradation (ERAD) to clear the protein accumulation (reviewed in [56]). Therefore, defective ER-Golgi trafficking could be the indirect cause of abnormal protein accumulation as well. Our findings that abnormal protein accumulation and CHOP expression, which is a sign of ER stress, are observed in *nur17* mice support both hypotheses. Since ER-stress is known to trigger signal transduction events that could induce cell death (reviewed in [57]), it may cause degeneration of PCs in *nur17* mice. However, we did not observe upregulation of another ER stress marker, KDEL, which is the protein sequence found in both BiP/Grp78 and Grp94. This result may indicate that abnormalities in *nur17* PCs do not affect the typical ER stress pathway. Alternatively, we may not be able to detect the increase in these proteins, because only small numbers of PCs may be experiencing it at a time, which would be consistent with the low number of CHOP-positive cells we observed in *nur17* mice. Nevertheless, our results provide evidence that the defect in the COPI complex leads to neurodegeneration, and suggest that ER abnormalities may be involved in the neurodegenerative mechanism. Recent discovery of Scy1-like1 (*Scyl1*) as a gene responsible for motor neuron and PC degeneration in mice also suggests a link between the COPI pathway and neurodegeneration [58], [59]. SCYL1 binds to and co-localizes with β-COP and regulates COPI-mediated retrograde ER-Golgi trafficking [59]. Together with our results, these findings support the notion that compromised COPI-mediated vesicle trafficking causes neurodegeneration.

In addition to abnormal protein accumulation and possible signs of ER-stress, *nur17* mice show neurofibrillary tangles (NFT) in the cerebellum that are commonly observed in a number of neurodegenerative disorders such as Alzheimer's Disease, Amyotrophic lateral sclerosis and Down's Syndrome (reviewed in [60]). Although ER stress and NFT are often observed together in many human neurodegenerative diseases [61]–[63], the relationship between these conditions is largely unknown. Our findings that a mutation in a component of the COPI complex, which regulates ER-Golgi vesicle trafficking, results in formation of NFT suggests a possible new mechanism underlying NFT formation. Impaired ER-Golgi vesicle trafficking could be the cause for NFT formation in this model. Further analysis of *nur17* mice should advance our understanding of the molecular mechanism causing the formation of NFT, which may be shared by a variety of neurodegenerative disorders.

Provided that the *Arcn1* gene is likely expressed ubiquitously in all cell types, it is intriguing that gross abnormalities have been detected only in the skin/hair and the cerebellum. It is possible that there is another protein that can compensate for the loss of ARCN1 in other cell types and tissues, although such protein has not been biochemically discovered to date. Alternatively, the affected tissues and cell types may be extraordinarily susceptible to minor changes in efficiency of COPI function. A mutation in another ubiquitously expressed protein known to function in ER-Golgi and intra-Golgi trafficking, TRAPPC6A, also leads to a tissue-restricted phenotype, pigment dilution, in mice [64]. Skin cells may be particularly sensitive to defects in these trafficking mechanisms. On the other hand, skin may be a unique tissue where subtle defects in intracellular trafficking can be visibly identified without the integrity of the skin being grossly affected, making it a great *in vivo* model system to investigate the mechanisms of intracellular trafficking.

## Materials and Methods

### Animals

All experiments were performed in accordance with the National Institute of Health Guide for the Care and Use of Laboratory Animals and were approved by the Animal Care and Use Committee at the University of Wisconsin-Madison.

At Baylor College of Medicine, ENU mutagenesis was performed using C57BL/6J males [25]. *nur17* mice were isolated, crossed to 129S6/SvEvTac three times and maintained in a mixed background of C57BL/6J and 129S6/SvEvTac.

After importing *nur17* mice to the University of Wisconsin-Madison, we maintained the *nur17* mutant allele and obtained the affected animals for characterization by repeating this mating scheme: *nur17/nur17* founder females were mated with wild-type 129S1/SvImJ mice. *nur17/+* F1 mice were intercrossed to obtain affected *nur17/nur17* mice. *nur17/nur17* female mice were again mated with 129S1/SvImJ.*nur17/+* or *+/+* littermate mice were used as control mice in the experiments.

129S1/SvImJ and AKR/J mice were obtained from The Jackson Laboratory.

Both 129S6/SvEvTac and 129S1/SvImJ strains are homozygous for the *A^w^* allele and display white-bellied agouti coat color. All *nur17* and control mice that were phenotyped in this study also displayed agouti coat color.

We replaced the EGFP sequence in the pCX-EGFP vector (kindly provided by Dr. Junichi Miyazaki; [65]) with the full length *Arcn1* cDNA and named it pCX-Arcn1. We used pCX-Arcn1 for the transgene construct for the rescue experiment after linearization with *BamHI* and *SalI* (New England Biolabs). The construct was micro-injected into pronuclei of FVB/NJ embryos to generate transgenic mice by the transgenic animal facility of the Biotechnology Center at the University of Wisconsin-Madison. We obtained two transgene-positive founders (2361 and 2368) and crossed them to *nur17* mice, and then intercrossed to obtain Tg-positive *nur17*/*nur17* mice.

### Southern blotting genotyping procedures

We identified Transgene (Tg)-positive clones by Southern-blot hybridization using *PstI* (New England Biolabs) digestion and probes specific to *Arcn1*. The southern blot differentiated the endogenous *Arcn1* and the transgene based on size of bands (1.05kb and 0.45kb for the transgene).

Tail DNA was prepared as described in [66]. ∼15µg of DNA was digested with the restriction enzyme *PstI* (New England Biolabs) and resolved by 0.8% agarose gel electrophoresis. DNA was transferred to Hybond N membrane (Amersham, GE Healthcare) using alkaline capillary transfer, crosslinked with UV (Spectroline). The membrane was prehybridized with prehybridization buffer (4xSSC, 1% milk powder, 1% SDS, 10× Denharts Reagent) and hybridized with probes labeled with [P32] dCTP (Perkin Elmer) by Rediprime II Random Prime Labeling System (Amersham, GE Healthcare) at 65°C using hybridization buffer [4xSSC, 1% milk powder, 1% SDS and 37µg/ml salmon sperm DNA (Sigma)]. The probe used for hybridization was PCR amplified from mouse brain cDNA using primers, mArcn1-F3 (5′ AAGCACCAGGATTTGGCGG 3′) and mArcn1-R16 (5′ TTACAGGATTTCGTATTTGTC 3′). Membranes were washed three times with wash buffer I (2xSSC and 0.1% SDS) and twice with wash buffer II (0.5xSSC and 0.1% SDS) for 15 min at 65°C for each wash. CL-X Posure Film (Thermo Scientific) was used to detect the hybridization signal.

### Northern blotting

The expression of the transgene was tested by a northern blot that differentiates the endogenously expressed *Arcn1* (4.4kb) and expressed transgene (2.2kb). Total RNA was extracted from mouse brains by Trizol (Invitrogen). mRNA was purified from total RNA by Oligotex mRNA Midi Kit (Qiagen). mRNA was resolved by formaldehyde agarose electrophoresis and transferred to Hybond N membrane (Amersham, GE Healthcare) using alkaline capillary transfer, crosslinked with UV (Spectroline). The membrane was prehybridized with Rapid-Hyb Buffer (Amersham) with 37µg/ml salmon sperm DNA (Sigma) and hybridized with probes labeled with [P32] dCTP (Perkin Elmer) by Rediprime II Random Prime Labeling System (Amersham, GE Healthcare) at 65°C using Rapid-Hyb Buffer (Amersham) with 37µg/ml salmon sperm DNA (Sigma). The probe used was the same described above for the Southern-blot; similarly, the membranes were washed and hybridization signal was detected as described above.

### Genetic mapping

To map the *nur17* gene, we performed a whole genome scan using F2 animals from mating (*nur17*×AKR/J) F1 mice. We initially used 80 microsatellite markers, which distinguish AKR alleles from both 129S1/SvImJTac and C57BL/6J alleles across the whole genome. All F2 animals were phenotyped by both coat color dilution and ataxia movement. Once the chromosomal locus on chromosome 9 was identified for the *nur17* mutation, additional F2 mice were collected and additional markers were used to further narrow the genetic region. We used two SNPs, D9SNP3 (SNP ID on NCBI: NES11330700) and D9SNP25 (SNP ID on NCBI: NES11338581), to differentiate AKR and *nur17* alleles.

### Genotyping

All genotyping was carried out by polymerase chain reaction (PCR). For *nur17* genotyping, PCR primers, mArcn1-F18 (5′ CCTCAAACTCAGAAATCCGC 3′) and mArcn1-R19 (5′ TTGGCATCAATCACTGGC 3′), were used for amplification of the wild type (WT) allele and *nur17* allele (470bp). *BsrI* (New England Biolabs) was used to digest the *nur17* allele specifically to generate two bands (392bp and 78bp).

### Immunohistochemistry

Littermate control (+/+, *nur17*/+) and *nur17* mice were deeply anesthetized with a mixture of ketamine and xylazine and perfused with 4% paraformaldehyde (PFA). The heads were immersed in 4% PFA overnight at 4°C, and the brain was dissected out. For paraffin sections, the brain was dehydrated in a graded ethanol series, cleared in xylene, and embedded in paraffin. Sections were cut at 6 µm, mounted on slides pretreated with Vectabond (Vector Laboratories). For cryostat sections, the brain was cryoprotected at 4°C in a series of sucrose gradients after dissection. Brains were embedded in optimal cutting temperature compound (OCT) (Sakura Finetek) and sectioned at 12µm thickness.

For immunohistochemistry, sections were blocked with 2% donkey serum and were incubated overnight with the primary antibody against Calbindin-D (Swant), Calbindin (Abcam), phosphorylated tau (abcam), hyperphosphorylated tau (Thermo Scientific) and CHOP (Santa Cruz Biotechnology). Sections were rinsed in PBS, and incubated with a 1∶200 diluted Alexa 488 conjugated secondary antibody (Invitrogen) and/or Cy3 conjugated secondary antibody (Jackson Immunoresearch) for 45 minutes at room temperature. All sections were imaged on an Eclipse E600 microscope (Nikon) using a SPOT camera (Spot Diagnostics).

### Electron microscopy

Littermate control (+/+, *nur17*/+) and *nur17* mice were anesthetized with ketamine/xylazine and perfused with 2% PFA and 2.5% glutaraldehyde in 0.1M phosphate buffer for 11 min. Cerebella were removed from the head and immersion fixed for 30 min. Then, cerebella were sectioned sagittally at 100µm intervals with a Vibratome. Sections of the cerebella were osmicated (1% osmium tetroxide) for 1 hr and washed in 0.1M phosphate buffer, dehydrated through an ascending series of ethanol and propylene oxide and embedded in Epon [25g Epon 812, 13g Dodecenyl Succinic Anhydride (DDSA), 12g Nadic Methyl Anhydride (NMA) and 1ml 2,4,6-tris(dimethylaminomethyl)phenol (DMP-30), Electron Microscopy Sciences]. Ultra-thin sections (70nm) were cut and stained with uranyl acetate and lead citrate. Sections were imaged on a Philips CM120 Scanning Transmission Electron Microscope.

### Gallyas-silver staining

The Gallyas-silver staining of the littermate control (+/+, *nur17*/+) and *nur17* cerebellum was performed as described in [67] in order to examine the existence of neurofibrillary tangles.

### Melanocyte culture

Mouse melanocytes were isolated from the skin on the back of the Day2 littermate control (+/+, *nur17*/+) and *nur17* mice and cultured. Fresh skin specimens were incubated in foreskin media (Dulbecco's modified minimal essential medium (DMEM) (Gibco) supplemented with 2% fetal bovine serum (Sigma-Aldrich), penicillin (100 units/ml, Sigma) and gentamicin (100 ug/ml) (Gibco) at 4°C overnight. The next day, the skin was washed three times with Hanks' balanced salt solution and excess fat was removed. The samples were cut into small pieces and incubated in 0.25% trypsin solution (Hyclone) supplemented with 3.86 mg/ml trypsin, from porcine pancreas (Sigma-Aldrich) at 4°C overnight. Epidermis was separated from the dermis and epidermal cells were suspended and cultured in Ham's F10 nutrient medium supplemented with 10% fetal bovine serum, 85 nmol/L 12-O-tetradecanoylphorbol-13-acetate (TPA) (Sigma-Aldrich), 0.1 mmol/L 3-isobutyl-1-methylxanthine (IBMX) (Sigma-Aldrich), 2.5 nmol/L cholera toxin (CT) (ICN Biochemicals) and penicillin (100 units/ml) (Gibco).

### Expression constructs and transfection of Neuro-2a cells

Ultimate ORF Human Clones for ARCN1 and coatomer protein complex, subunit beta 1 (COPB1 or beta-COP) in Gateway entry vector (Invitrogen; ARCN1: IOH43520, COPB: IOH27122) were purified using a QIAprep Spin Miniprep Kit (Qiagen) after culturing on the LB agar plate containing 10µg/mL of kanamycin and in the LB liquid medium. pcDNA-DEST53 (GFP-attR1-Cm^R^-ccdB-attR2; Invitrogen) was used as the destination vector for ARCN1, and pcDNA™/V5-DEST (attR1-ccdB-Cm^R^-attR2-V5 epitope; Invitrogen) was used as the destination vector for COPB1. The LR recombination reaction between the entry clone and a destination vector was carried out using LR Clonase Enzyme (Invitrogen) according to the protocols recommended in the product manual. The expression constructs were then purified using a QIAfilter Plasmid Midi Kit (Qiagen).

Mouse Neuro-2a cells (ATCC) were cultured in DMEM with high glucose (Gibco), supplemented with 10% FBS (Sigma). The ARCN1 and COP1B expression constructs were transfected into Neuro2A cells using SuperFect Transfection Reagent (Qiagen) following the manufacture's protocol and cultured for 24 hrs.

### Immunocytochemistry

Cells were cultured on coverslips and were fixed by 4% PFA for 10 min at 4°C. Cells were permeabilized using 0.5% Triton-X in PBS for 30 min followed by blocking in 2% normal donkey serum for 30 min. Then the cells were incubated with 1∶200 diluted primary antibody against ARCN1 (Novus Biologicals), 58K Golgi protein (58K-9) (Abcam), KDEL (Abcam), β-COP (Sigma), GFP (Synaptic System) and V5 (Invitrogen) in PBS with 0.1% Triton-X for 1 hour at room temperature. Cells were washed in PBS and then incubated with 1∶200 diluted Alexa 488 conjugated secondary antibody (Invitrogen) and Cy3 conjugated secondary antibody (Jackson Immunoresearch) for 45 min at room temperature. All immunocytochemistry slides were imaged on a Zeiss 510 confocal laser scanning system and Axio Imager microscope using LSM 510 software (release 4.2) (Carl Zeiss MicroImaging, Inc).

### Pulse-chase labeling

Melanocytes grown in a 5cm culture dish until 90–100% confluency were starved in DMEM without methionine (Cellgro) supplemented with 10% fetal bovine serum (Sigma-Aldrich), penicillin (100 units/ml) (Sigma) for 2 hours. Each plate of cells was labeled with 50 uCi [^35^S] methionine (EXPRE35S35S protein labeling mix; New England Nuclear) for 10 min, washed twice, and chased in normal growth medium for different periods of time. Cells were harvested, washed twice with ice-cold PBS, and lysed in lysis buffer (PBS with 0.1% triton X). The lysates were cleared by centrifugation at 15,000 g for 10 min at 4°C, and incubated with mAb TA99 (1∶10 dilution, homemade) at 4°C for overnight followed by rabbit anti-mouse IgG coupled to protein A Sepharose (1∶10 dilution) for 2 hours at 4°C. Immunoprecipitates were washed with 10 mM Tris/HCl, pH 7.5, 0.15 M NaC1, 5 mM EDTA, and 1% NP-40 (TNEN), followed by TNEN containing 0.5 M NaCI, and finally with distilled water. For Endo H digestion, the immunoprecipitates were dissociated by suspending in 0.2% SDS, heated for 5 min at 100°C, and diluted with 25 ul 0.1 M sodium citrate buffer, pH 5.5, Endo H, 50 mU/ml. The reaction mixture was layered over with 50ul toluene and digestion was carried out at 37°C for 18–24 h. Littermate control (+/+, *nur17*/+) tubes were treated similarly, except that equal volume of 0.1 M sodium citrate buffer, pH 5.5, was added instead of Endo H. Proteins were analyzed by 9% SDS-PAGE. Radioactive protein bands were visualized by fluorography. The proportion of Tyrp1 without complex N-linked glycans against the total protein was determined by scanning densitometry using the gel analysis function of the ImageJ software (http://rsb.info.nih.gov/ij). The signals at the lower band position were quantified for Tyrp1 without complex N-linked glycans, and all the signals spanning the lower and higher band positions were quantified for the total protein.

### Statistical analysis

Student's t-test was performed for statistical comparison of the weight of mice, the number of cells positive for Gallyas staining or the CHOP1 staining between littermate control (+/+, *nur17*/+) and *nur17* mice and the latency to fall in the rotarod analysis. Two-way analysis of variance (ANOVA) was performed for the statistical comparison of the proportion of protein without complex N-linked glycans in the pulse-chase experiment. GraphPad Prism software (GraphPad software, Inc.) was used for statistical analysis and to create all graphs reporting numerical values. * p<0.05, ** p<0.01.

### Rotarod analysis

Mice that were of the genotype of +/+ or *nur17*/+ TG− (n = 5), *nur17*/*nur17* TG− (n = 5) and *nur17*/*nur17* TG+ (n = 5) at 5–8 months of age were subjected to rotarod analysis. Mice were first exposed to a training period at constant speed to familiarize them with the rotarod apparatus (Ugo Basile). For the testing, the rotorod was gradually accelerated (1 rpm/s) from 4 rpm over the course of 3 min. The time was recorded when a mouse fell from the device (latency to fall). After all mice fell from the rotorod, the group was given a rest period and then reloaded on the device. The testing was performed a total of 3 times for each mouse. Values from the three trials for each mouse were averaged to give a single score.

## Supporting Information

Figure S1 Genotyping and transgene expression in the transgenic mice. (A) Southern blot for the transgene. The mice from line 2361 and line 2368 with the transgene exhibit two extra bands (1.06kb and 0.45kb), which are marked by arrowheads. (B) Northern blot analysis to test the expression of transgene. The mice from line 2361, control and line 2368 all exhibit the band for endogenous *Arcn1* (4.1kb), while only line 2361 exhibits the band from the transgene (2.2kb, arrowhead).
(2.29 MB TIF)

Figure S2 Immunofluorescence for KDEL in the cerebellum. Positive staining is observed in both control (left) and nur17 PCs. Nuclei are stained with DAPI (blue). Scale bar: 10 µm.
(1.65 MB TIF)

Figure S3 Comparison of the subcellular localization of ARCN1. Primary culture of wild-type control (A,C,E) and nur17 (B,D,F) mouse melanocyte are all labeled with anti-ARCN1 (green) indicating the localization of ARCN1 and anti-Golgi protein (red) (A,B); anti-KDEL (red) (C,D) and anti-βCOP (red) (E,F). Nuclei are stained with DAPI (blue). Merged pictures are shown and high magnification pictures are shown in the right panel. Scale bar for low magnification: 10 µm. Scale bar for high magnification: 5 µm.
(2.18 MB TIF)
