# Supplementary figures and images for: Mutation in Archain 1, a Subunit of COPI Coatomer Complex, Causes Diluted Coat Color and Purkinje Cell Degeneration
Source: PLoS Genet. 2010 May 20;6(5):e1000956. doi: 10.1371/journal.pgen.1000956 (PMC2873907; doi:10.1371/journal.pgen.1000956)

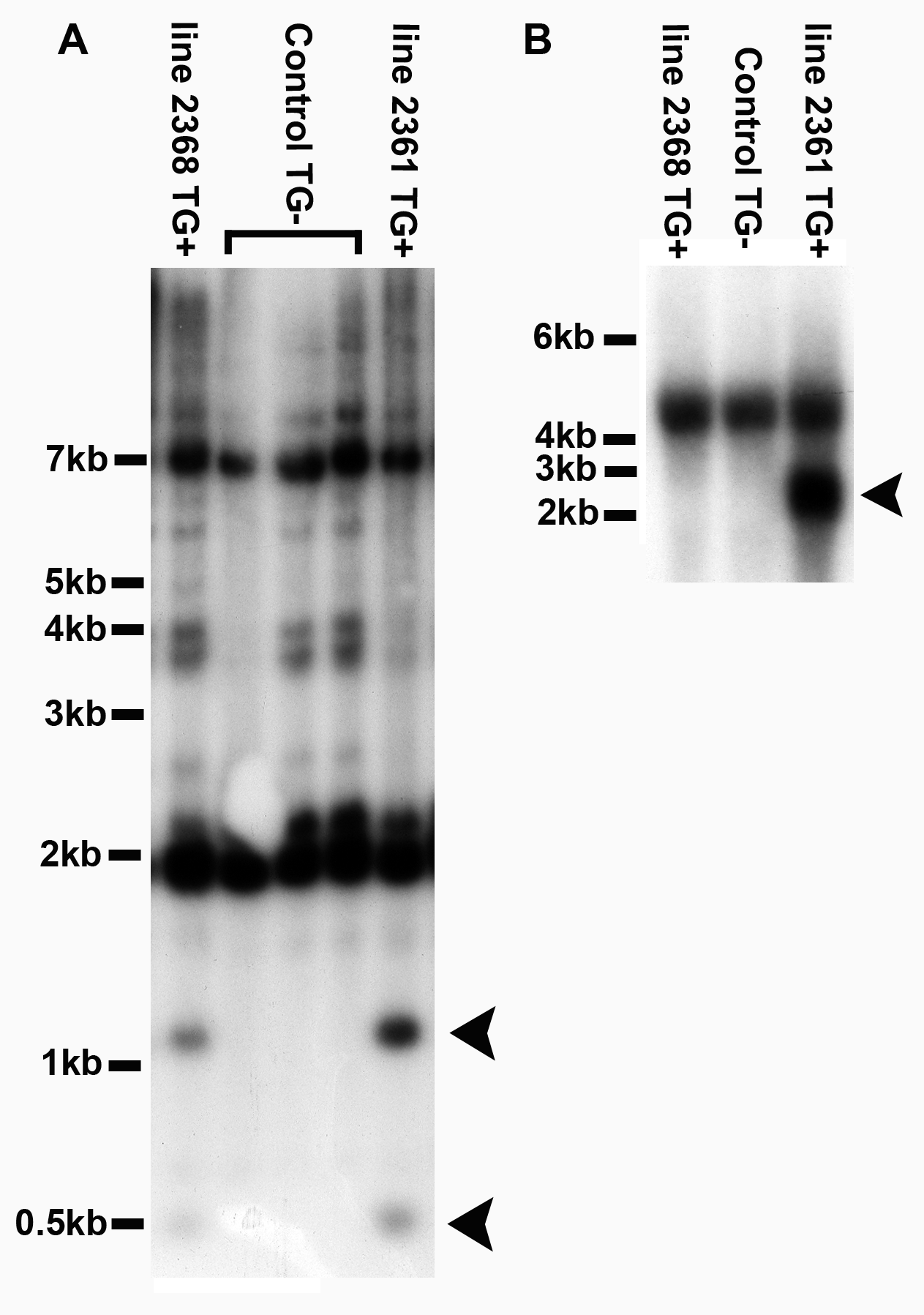

Supplement: Figure S1 — Genotyping and transgene expression in the transgenic mice. (A) Southern blot for the transgene. The mice from line 2361 and line 2368 with the transgene exhibit two extra bands (1.06kb and 0.45kb), which are marked by arrowheads. (B) Northern blot analysis to test the expression of transgene. The mice from line 2361, control and line 2368 all exhibit the band for endogenous Arcn1 (4.1kb), while only line 2361 exhibits the band from the transgene (2.2kb, arrowhead). (2.29 MB TIF) [file pgen.1000956.s001.tif]

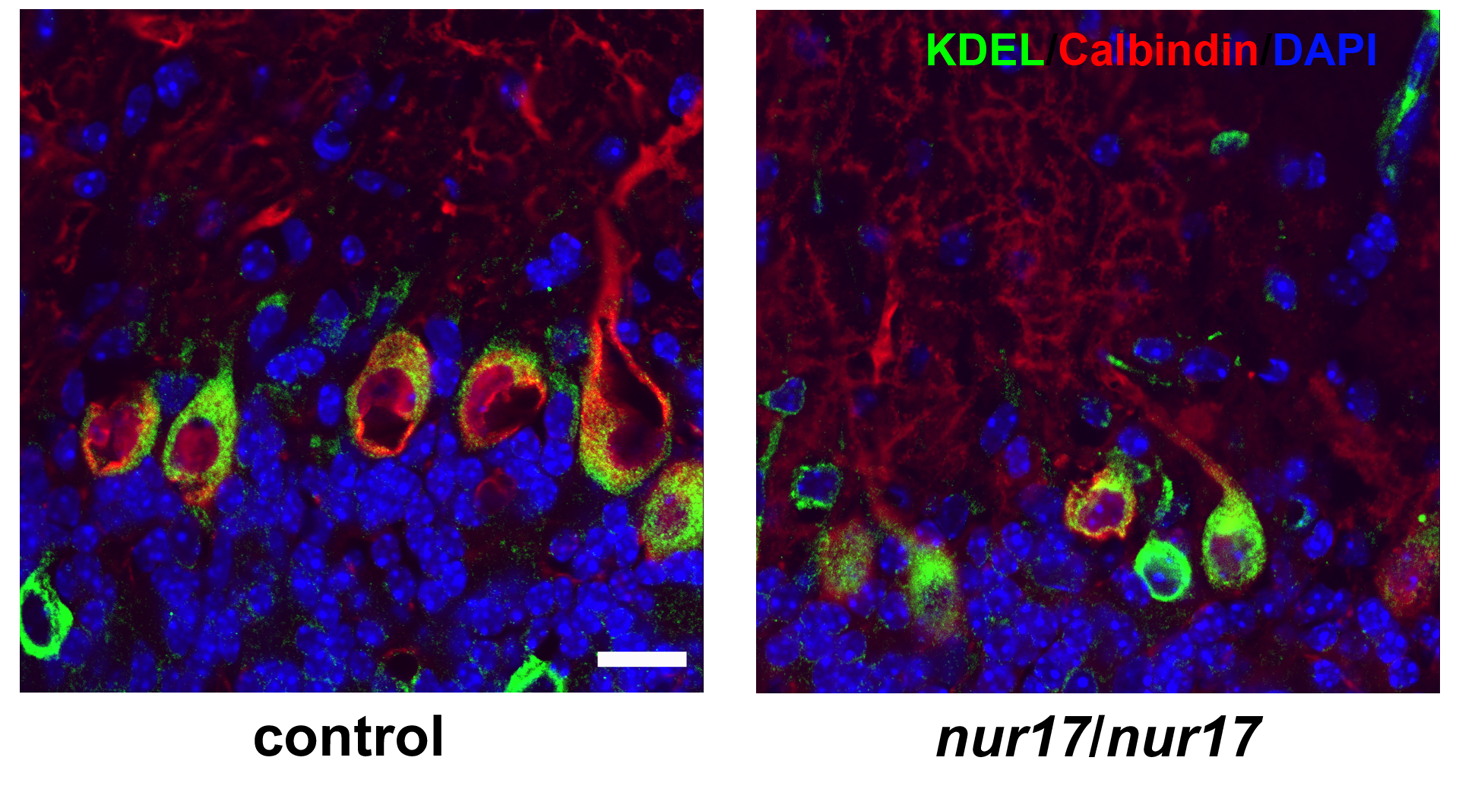

Supplement: Figure S2 — Immunofluorescence for KDEL in the cerebellum. Positive staining is observed in both control (left) and nur17 PCs. Nuclei are stained with DAPI (blue). Scale bar: 10 µm. (1.65 MB TIF) [file pgen.1000956.s002.tif]

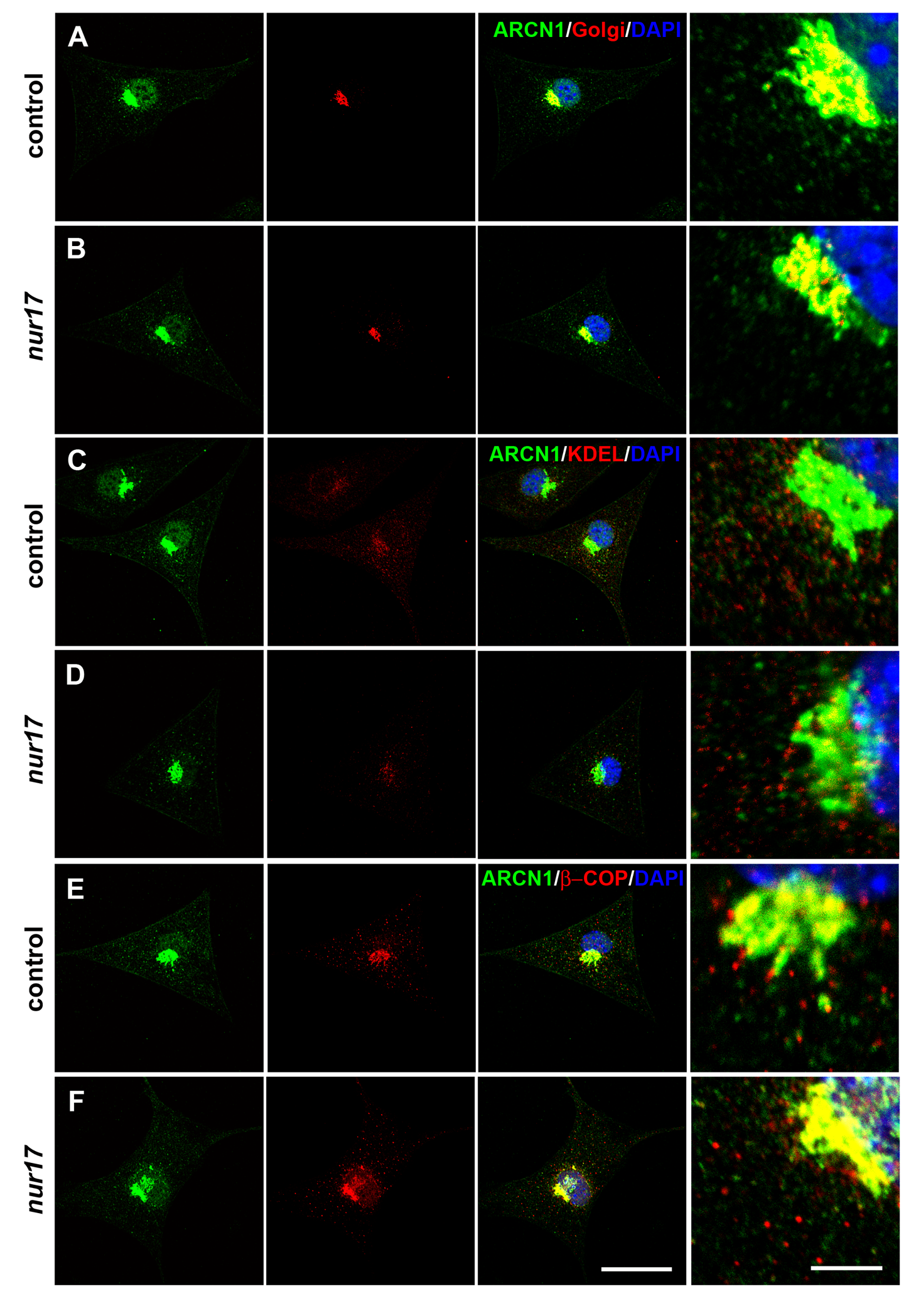

Supplement: Figure S3 — Comparison of the subcellular localization of ARCN1. Primary culture of wild-type control (A,C,E) and nur17 (B,D,F) mouse melanocyte are all labeled with anti-ARCN1 (green) indicating the localization of ARCN1 and anti-Golgi protein (red) (A,B); anti-KDEL (red) (C,D) and anti-βCOP (red) (E,F). Nuclei are stained with DAPI (blue). Merged pictures are shown and high magnification pictures are shown in the right panel. Scale bar for low magnification: 10 µm. Scale bar for high magnification: 5 µm. (2.18 MB TIF) [file pgen.1000956.s003.tif]
